# Phytosterol supplementation in the treatment of dyslipidemia in children and adolescents: a systematic review

**DOI:** 10.1590/1984-0462/2021/39/2019389

**Published:** 2020-11-11

**Authors:** Luisa Montone Mantovani, Camila Pugliese

**Affiliations:** a Universidade de São Paulo, São Paulo, SP, Brazil.

**Keywords:** Phytosterols, Dietary supplements, Dyslipidemias, Hypercholesterolemia, Child, Fitoesteróis, Suplementos nutricionais, Dislipidemia, Hipercolesterolemia, Criança

## Abstract

**Objective:**

To carry out a systematic review on the effects of phytosterol supplementation on the treatment of dyslipidemia in children and adolescents.

**Data sources:**

Review in the SciELO, Lilacs, Bireme, PubMed and Web of Science databases, with no time limit. Descriptors: phytosterols or plant sterols and dyslipidemias, hypercholesterolemia, cholesterol, children, adolescent, in English and Portuguese. The articles included were published in Portuguese, English or Spanish and evaluated the effect of phytosterol supplementation in pediatric patients with dyslipidemia. Documents that involved adults or animals, review papers, case studies and abstracts were excluded. Two authors performed independent extraction of articles. Of 113 abstracts, 19 were read in full and 12 were used in this manuscript.

**Data synthesis:**

Phytosterol supplementation to reduce cholesterol levels has been shown to be effective in reducing LDL-cholesterol levels by approximately 10%, with reductions above 10% in LDL-cholesterol levels observed after 8 to 12 weeks of intervention. Studies have not shown significant changes in HDL-cholesterol and triglyceride levels. Based on the absence of adverse effects, its use seems to be safe and of good tolerance in children and adolescents.

**Conclusions:**

Phytosterol supplementation seems to be of great therapeutic aid for the treatment of hypercholesterolemia in children and adolescents. Further studies assessing the long-term effect of phytosterol supplementation are necessary.

## RESUMO

**Objetivo:**

Realizar uma revisão sistemática sobre os efeitos da suplementação de fitoesteróis no tratamento da dislipidemia em crianças e adolescentes.

**Fontes de dados:**

Revisão nos bancos SciELO, Lilacs, Bireme, Pubmed e Web of Science, sem limite de tempo. Descritores: *phytosterols or plant sterols*, *dyslipidemias*, *hypercholesterolemia*, *cholesterol*, *children*, *adolescent*, nas línguas inglesa e portuguesa. Os artigos incluídos foram publicados nos idiomas português, inglês ou espanhol e avaliaram o efeito da suplementação de fitoesteróis em pacientes pediátricos com dislipidemia. Estudos que envolviam adultos ou animais, trabalhos de revisão, estudos de caso e resumos foram excluídos. A extração independente de artigos foi realizada por dois autores. Do total de 113 resumos, 19 foram lidos na íntegra, e 12 utilizados neste manuscrito.

**Síntese de dados:**

A suplementação de fitoesteróis para a redução dos níveis de colesterol mostrou-se eficiente, de forma a promover a redução de aproximadamente 10% dos níveis de LDL-colesterol, sendo observadas reduções acima de 10% em 8 a 12 semanas de intervenção. Os estudos não mostraram alterações significantes nos níveis de HDL-colesterol e triglicérides. Com base na ausência de efeitos adversos, seu uso parece ser seguro e de boa tolerância em crianças e adolescentes. **Conclusões:** A suplementação com fitoesteróis parece ser de grande auxílio terapêutico para o tratamento da hipercolesterolemia em crianças e adolescentes. São necessários mais estudos que avaliem o efeito em longo prazo da suplementação de fitoesteróis.

## INTRODUCTION

Dyslipidemia in children and adolescents represents a determinant risk factor for atherosclerosis and can contribute with coronary disease in adulthood.^1^ It has been established that fat cells are present in the aorta of individuals at the age of 10, and in coronary arteries at the age of 20, and that the progression of fatty streaks occurs after the age of 15.^2^

Dyslipidemia is a metabolic disorder characterized by abnormal concentrations of lipids and/or lipoproteins in the blood.^2^ It is defined by the elevation in total cholesterol (TC) levels or low-density lipoproteins (LDL) and/or triglycerides (TG), and/or the reduction of high-density lipoproteins (HDL).^3^ Besides, it is known that the elevation of LDL-cholesterol levels is a risk factor established for cardiovascular disease.^1^

Lipid disorders may have primary (of genetic origin) or secondary (resulting from inappropriate lifestyle, some morbid conditions or medication) causes.^4^ Therefore, dyslipidemia in children is multifactorial, and may be associated with environmental and behavioral factors and with obesity, except in cases of genetic etiology, such as familial hypercholesterolemia (FH).^1^

The prevalence of dyslipidemia in the juvenile age group in Brazil ranges from 24 to 40%.^2^ The estimation is that, in the world, there are over 10 million individuals with FH; however, less than 10% have a known diagnosis of FH, and less than 25% receive lipid lowering treatment.^5^

The I Guideline for preventing atherosclerosis in childhood and adolescence recommends the pharmacological lipid lowering treatment for children aged more than 10 years, and proposes changes in dietary pattern and in the lifestyle of children older than two years of age, which include a diet with reduced levels of saturated fat and cholesterol, and practice of physical activity.^6^ Adequate dietary changes and an active lifestyle help to reduce the cardiovascular risk factors and should be encouraged, but in case there are no responses to such interventions, and in case it is not possible to recommend pharmacological treatment, the use of phytosterols can be considered.^3^

Plant sterols and stanols are also known as phytosterols, which are bioactive components whose structure is similar to that of cholesterol; sterols are the unsaturated forms (sitosterol and campesterol), and stanols are the saturated derivatives (sitostanol and campestanol).^4^ Their main role is to reduce LDL-cholesterol by inhibiting the intestinal absorption of cholesterol.^7^ They are naturally found in fruit, vegetables, vegetable oils, nuts and seeds.^8^ The intake of phytosterols through natural sources ranges from 200 to 400 mg per day in frequent diets.^9^ These items can also be added to foods such as margarine, juice, yogurt and cereals. Besides the use in enriched foods, it is also possible to supplement them.^2^

The inverse correlation between the frequent intake of phytosterols in the diet and serum levels of cholesterol or LDL-cholesterol^4^ has been proven, and their supplementation is recommended by several guidelines.^3^ ^,^ ^4^ ^,^ ^10^ ^,^ ^11^ However, their use is usually addressed to children with primary dyslipidemia; there is no agreement as to the dose and safety of this practice for all patients with dyslipidemia. Therefore, this review proposes the investigation of the effects of supplementation of phytosterols in the treatment of dyslipidemia in children and adolescents.

## METHOD

This review was carried out based on the recommendations in the Preferred Reporting Items for Systematic Reviews and Meta-Analyses^12^, in the following databases: SciELO, Lilacs, Bireme, PubMed and Web of Science. The descriptors used were *phytosterols or plant sterols*, *dyslipidemias*, *hypercholesterolemia*, *cholesterol*, *children*, *adolescent*, in English and in Portuguese. The bibliographic research was based on the question: “What are the effects of phytosterol supplementation on the treatment of dyslipidemia in pediatric patients?”, which was based on the Population, Intervention, Comparison, Outcome model.^13^

Inclusion criteria were clinical trials in English, Spanish and Portuguese, which assessed the effect of phytosterol supplementation in pediatric patients with dyslipidemia. We also considered the studies referred to by selected articles which met the inclusion criteria. Documents that did not concern the purpose of this study, involving adults or animals, case reviews, case studies, abstracts and analyses that did not include samples with dyslipidemia were excluded. There was no limitation as to the year of publication of the articles.

The identification of the articles was first carried out through the analysis of the title to exclude repeated articles, or the ones that did not contemplate the predefined criteria. Afterwards, the remaining abstracts were evaluated regarding their adaptation to the inclusion and exclusion criteria. Studies presenting the predetermined criteria were fully acquired for a detailed analysis and data extraction. The selected articles were read by two evaluators who decided, independently, about their inclusion based on the eligibility criteria. Any divergence about the selection of articles was decided consensually.

The selected studies had the following characteristics:

- Characteristics of the study participants (including age, type of dyslipidemia and method of diagnosis), and inclusion and exclusion criteria of the study.
- Type of intervention (including type of phytosterol, dose, duration, administration and food vehicle).
- Main results (including serum cholesterol reduction and lipid fractions, time of follow-up and unwanted effects of the treatment).

Then, a synthesis and a critical analysis were carried out considering the studies.

To verify the validity of the clinical trials, the following were determined: sample size, loss extension in the follow-up of participants in the studies and blinded patients, health professionals, data collectors and evaluators of results.

## RESULTS

**Figura 1 -:**
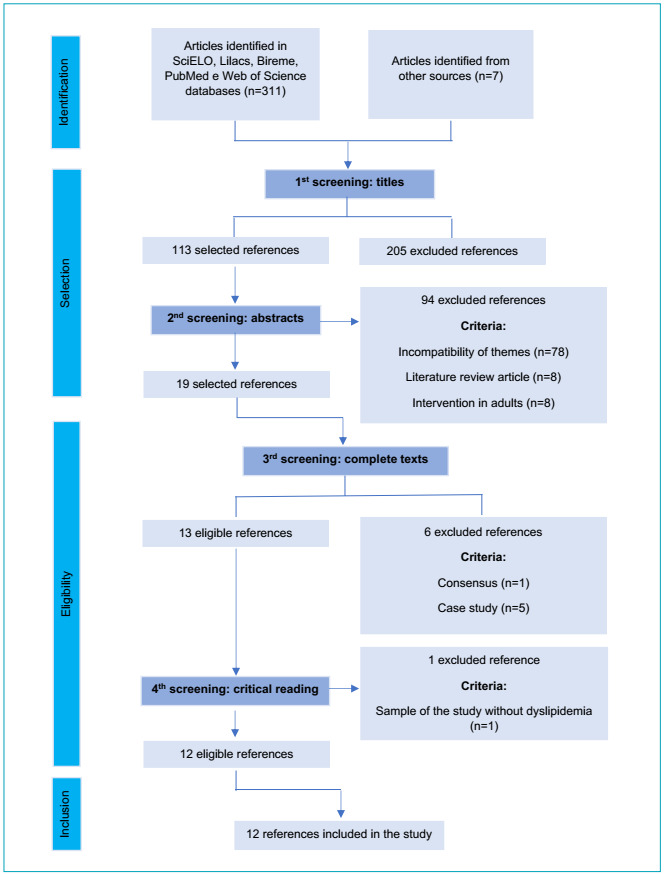
Flow diagram showing the stages of identification, selection, eligibility and inclusion of references.

Initially, 311 studies were identified in the electronic databases. After refinement by title, 113 abstracts were selected for analysis. The potentially relevant studies were fully acquired for a detailed analysis of the eligibility criteria. Additionally, seven studies identified in other reference lists of the selected articles, and related to the theme, were included. Finally, the descriptive synthesis was composed of 12 references, whose periods of publication were between 1993 and 2017.^14^ ^,^ ^15^ ^,^ ^16^ ^,^ ^17^ ^,^ ^18^ ^,^ ^19^ ^,^ ^20^ ^,^ ^21^ ^,^ ^22^ ^,^ ^23^ ^,^ ^24^ ^,^ ^25^ Figure 1 presents the flow diagram of the stages of data search and selection.

All of the selected studies are randomized^15^ ^,^ ^17^ ^,^ ^18^ ^,^ ^19^ ^,^ ^20^ ^,^ ^25^ and non-randomized^14^ ^,^ ^16^ ^,^ ^21^ ^,^ ^22^ ^,^ ^23^ ^,^ ^24^ clinical trials published in English. Ten of them were carried out in Europe,^14^ ^,^ ^15^ ^,^ ^16^ ^,^ ^17^ ^,^ ^18^ ^,^ ^19^ ^,^ ^20^ ^,^ ^22^ ^,^ ^23^ ^,^ ^24^ one in Japan^21^, and one in Brazil.^25^ All randomized studies had an initial double-blind period. The loss in participants’ follow-up was described as: non-adherence to the diet,^22^ ^,^ ^24^ ^,^ ^25^ personal reasons not related to the study,^17^ ^,^ ^22^ ^,^ ^25^ changes in pubertal stage,^24^ palatability of the product or nausea,^23^ difficulty with product intake.^20^ ^,^ ^22^ Table 1 exposes a brief description of all references according to the following items: author(s), year of publication, design, methods and conclusion.

**Table 1:** Selected articles, according to author(s), year, design, methods and conclusion.

Author(s), year	Design	Methods		Conclusion
		Type of dyslipidemia	Patients	
Becker et al., 1993^14^	Clinical trial	FH	n=9 (9-14 years old)	Sitostanol supplementation, even in doses inferior to sitosterol, was significantly more effective in the reduction of LDL-c, and its use can be indicated for the treatment of FH in children.
Gylling et al., 1995^15^	Double-blind randomized clinical trial	FH	n=15 (2-15 years old)	In a balanced diet, the use of sitostanol-ester margarine seems to be effective and safe for the treatment of hypercholesterolemia in children with FH.
Vuorio et al., 2000^16^	Clinical trial	FH	n=24 (3-13 years old)	The use of stanol-ester margarine has proven to be effective and safe for lipid lowering treatment in children.
Amundsen et al., 2002^17^	Double-blind randomized crossover clinical trial	FH	n=38 (7-12 years old)	In a balanced diet, sterol supplementation induces the reduction of LDL-c, without adverse effects, in children with FH.
Ketomãki et al., 2003^18^	Double-blind randomized crossover clinical trial	FH (n=17) Hypercholesterolemia** (n=6)	n=23 (2-9 years old)	Stanol and sterol esters reduce the concentrations of cholesterol in the plasma.
Amundsen et al., 2004^19^	Double-blind randomized controlled crossover clinical trial	FH	n=37 (7-13 years old)	Sterol supplementation such as spread is efficient to reduce cholesterol in children with FH in a controlled diet, and such a reduction is maintained for six posterior months.
Jakulj et al., 2006^20^	Double-blind randomized placebo-controlled clinical trial	FH	n=41 (7-12 years old)	The intake of stanols can be a beneficial and safe strategy, well accepted for the reduction of LDL-c levels in children with FH.
Matsuyama et al., 2007^21^	Clinical trial	Hyperlipidemia Type IIa (n=7) Hyperlipidemia* (n=8) FH (n=7)	n=22 (6-17 years old)	Phytosterols are able to reduce cholesterol in children with hyperlipidemia.
Guardamagna et al., 2011^22^	Clinical trial	Heterozigous FH (n=29) Combined FH (n=11) Hypercholesterolemia* (n=12)	n=52 (8-16 years old)	The daily consumption of sterol produces favorable changes in lipid profile, reducing LDL-c.
Garaiova et al., 2013^23^	Clinical trial	Hyperlipidemia*	n=25 (11-17 years old)	The combined emulsion of sterol plants, fish oil and vitamin B can modulate the lipid profile in children and adolescents with hypercholesterolemia.
Garoufi et al., 2014^24^	Clinical trial	Hypercholesterolemia*	n=59 (4.5-15.9 years old)	Sterol supplementation can be beneficial for the treatment of hypercholesterolemia in children; not only regarding LDL-c levels, but also more atherogenic particles.
Ribas et al., 2017^25^	Double-blind randomized clinical trial	Hypercholesterolemia **	n=25 (6-19 years old)	Plant sterol supplementation is effective and safe for the treatment of dyslipidemia in children.

FH: familial hypercholesterolemia; LDL-c: low density lipoprotein-cholesterol; TG: triglycerides; CRP: C-reactive protein; *undetermined etiology; **etiology without genetic cause.

In total, 370 children aged between 2 and 19 years were included, and the sample size variation of the studies was from 9 to 59 participants. As to the type of dyslipidemia, nine studies included children/adolescents with FH,^14^ ^,^ ^15^ ^,^ ^16^ ^,^ ^17^ ^,^ ^18^ ^,^ ^19^ ^,^ ^20^ ^,^ ^21^ ^,^ ^22^ and six with dyslipidemia without genetic cause or with no determined etiology.^18^ ^,^ ^21^ ^,^ ^22^ ^,^ ^23^ ^,^ ^24^ ^,^ ^25^ No study included or reported the inclusion of children/adolescents on lipid lowering medication. Three studies included patients using medication for asthma or allergy^17^ ^,^ ^19^ ^,^ ^20^ and one using medication for attention deficit hyperactivity disorder.^20^ A clinical trial excluded children/adolescents diagnosed with phytosterolemia,^14^ and five excluded patients with chronic conditions.^18^ ^,^ ^22^ ^,^ ^23^ ^,^ ^24^ ^,^ ^25^ Two studies asked the students to exclude the intake of products containing plant stanols or sterols before the beginning of the analysis in order to prevent bias.^18^ ^,^ ^25^

In two clinical trials,^17^ ^,^ ^19^ part of the participants used fish oil (cod-liver oil or omega 3 polyunsaturated fatty acids) and vitamin supplement containing retinol, tocopherol and vitamin D (none contained carotenoids). Despite being instructed to consume the same dose of the medication/supplement during the entire study, there may be bias in the outcome of the analysis of serum levels of fat-soluble vitamins and antioxidants.

During the study period, the children and adolescents’ diet contained low levels of saturated fat and cholesterol, established by the respective authors.^14^ ^,^ ^15^ ^,^ ^16^ ^,^ ^17^ ^,^ ^18^ ^,^ ^19^ ^,^ ^20^ ^,^ ^22^ ^,^ ^23^ ^,^ ^24^ ^,^ ^25^ Only in the study by Matsuyama et al.^21^, participants did not undergo dietary restrictions; however, they were advised to maintain their eating habits during the study.

The intervention in the studies regarding the type of phytosterol, dose, duration and food vehicles used is represented in Table 2. In all of the selected clinical trials, the evaluated primary outcome was the reduction in the serum concentration of LDL-cholesterol.

**Table 2:** Randomized clinical trials evaluating the effects of phytosterol supplementation on cholesterolemia.

Author(s), year	Type of phytosterol	Dose (g/day)	Duration (in weeks)	Vehicle	↓LDL-c (%)	**p-value (*versus* control group)**	↓TC (%)	**p-value (*versus* control group)**
Becker et al., 1993^14^	Sterols Stanols	6 1.5	12 12 and 28	Tablets	19.5 33.2 and 29.2	<0.01	17.1 25.7 and 23.5	<0.01
Gylling et al., 1995^15^	Stanols	3	6	Margarine	15	<0,.01	10.6	<0.01
Vuorio et al., 2000^16^	Stanols	2.24	12	Margarine	17.9	<0.001	13.6	<0.001
Amundsen et al., 2002^17^	Sterols	1.6	8	Spread*	10.2	<0.01	7.4	<0.01
Ketomãki et al., 2003^18^	Sterols Stanols	2	5	Spread*	9 12	<0.001	6 9	<0.01
Amundsen et al., 2004^19^	Sterols	1.2	8	Spread*	11.4	<0.001	9.1	<0.001
Jakulj et al., 2006^20^	Stanols	2	4	Skimmed yogurt	9.2	<0.001	7.5	<0.001
Matsuyama et al., 2007^21^	Sterols	0.4	16	Bread	All: 6.3 FH: 11.2	<0.05	All: 2.2 FH: 6	All: NS FH: <0.05
Guardamagna et al., 2011^22^	Sterols	1.6 to 2	12	Skimed yogurt	FH: 12.4 UDH: 16	<0.05	FH: 10.2 UDH: 13	<0.05
Garaiova et al., 2013^23^	Sterols	1.3	16	Emulsion	8.4	<0.05	7.7	<0.05
Garoufi et al., 2014^24^	Sterols	2	48	Skimmed yogurt	13	<0.001	9.4	<0.001
Ribas et al., 2017^25^	Sterols	1.2	8	Skimmed milk	10.2	<0.01	5.9	NS

LDL-c: low density lipoprotein-cholesterol; TC: total cholesterol; FH: familial hypercholesterolemia; NS: not significant; UDH: undetermined hypercholesterolemia; *pasta.

The studies did not show significant changes in levels of HDL-cholesterol and TG, except for the results found in a single clinical trial,^14^ which observed a 12% reduction (p<0.05) in levels of HDL-cholesterol, and significant increase p<0.05) of TG after three to seven months of supplementation, but remained in the normality range.

 Only two studies included the analysis of the effect of phytosterol supplementation after the intake period. The study by Matsuyama et al.^21^ verified a significant increase of TC and LDL-cholesterol levels after four months (p<0.05). On the other hand, Amundsen et al.^19^ observed that the efficiency in the reduction of cholesterol was maintained in the six following months.

After the intervention, some of the clinical trials observed reduction in the plasmatic concentration of hydrocarbon carotenoids (β-carotene,^14^ ^,^ ^16^ ^,^ ^17^ ^,^ ^19^ α-carotene^14^ ^,^ ^19^ and lycopene^17^), α-tocopherol^16^ ^,^ ^23^ and retinol.^23^ Other studies identified increase in α-tocopherol^19^ and retinol.^17^ ^,^ ^19^ The other studies did not verify changes in levels of β-carotene,^23^ y-tocopherol^23^ and retinol.^16^

Only two studies^20^ ^,^ ^22^ identified side effects in phytosterol supplementation, and the most recurrent complaint, in both studies, was abdominal discomfort in six children, compared to eight in the placebo group and one in the intervention group, respectively. The other studies did not report side effects.

## DISCUSSION

Phytosterol supplementation for the reduction of cholesterol levels, according to the clinical trials mentioned in this study, has shown to be efficient, in order to promote the reduction of approximately 10% of LDL-cholesterol levels. Most of the clinical trials considered presented a sample of patients with FH.^14^ ^,^ ^15^ ^,^ ^16^ ^,^ ^17^ ^,^ ^18^ ^,^ ^19^ ^,^ ^20^ ^,^ ^21^ ^,^ ^22^ However, among those that included children and adolescents with hypercholesterolemia of undetermined etiology or without genetic causes also obtained beneficial results about cholesterolemia.^18^ ^,^ ^21^ ^,^ ^22^ ^,^ ^23^ ^,^ ^24^ ^,^ ^25^

The subjacent mechanism to this hypocholesterolemic effect is the reduced absorption of cholesterol of the intestinal lumen in the circulation due to a competition between plant stanols/sterols and intestinal cholesterol by the incorporation in mixed micelles. Phytosterols are more hydrophobic than cholesterol, so, they group better inside the micelle.^26^ Therefore, they prevent the incorporation of cholesterol in the micelles, reducing its bioavailability and the flow of cholesterol of the intestinal lumen to the circulation, and increasing cholesterol synthetized by the liver.^27^ The free cholesterol that is not incorporated inside the micelles is eliminated in the feces, resulting in the reduced absorption of cholesterol in the enterocytes. The effect of reduced absorption and increased synthesis of cholesterol is a reduction in serum concentrations of LDL.^28^ Even though there are many studies about phytosterols, its hypocholesterolemic action has not been completely clarified.^29^

The average dose of administered phytosterols was of about 2.1 g/day (variation of 0.4 to 0.6 g/day) in children aged between 2 and 19 years. However, the Brazilian Society of Cardiology suggests that the use of phytosterols should be part of the changes in lifestyle, and is indicated for children aged more than 5 years with FH, as approved for use in Brazil.^4^ Therefore, the I Brazilian Guideline of Familial Hypercholesterolemia recommends the intake of 1.2 to 1.5 g per day in children with heterozygous FH.^3^ The European Society of Cardiology and the European Atherosclerosis Society,^10^ as well as the Expert Panel on Integrated Guidelines for Cardiovascular Health and Risk Reduction in Children and Adolescents,^11^ suggest, as a support measure, the consumption of 2 g of sterols / stanols per day in children with FH. On the other hand, the consensus of the Brazilian Association of Nutrology proposes that obese children and adolescents with dyslipidemia receive 1.6 g of phytosterol every day.^2^ Besides, the assumption is that all children and adolescents with dyslipidemia without indication for pharmacological treatment would benefit from the use of phytosterol supplementation to reduce levels of TC and LDL, as observed in this study.

The selected clinical trials used the administration of plant sterols or stanols, or both, for intervention. The main phytosterols used were sitosterol and sitostanol. About the different subclasses of phytosterols, the studies that compared the efficacy of plant stanols and plant sterols verified that even though both have led to reduction in LDL-cholesterol, a lower dose of plant stanol seems to be able to reduce LDL more effectively.^14^ ^,^ ^18^ However, a meta-analysis carried out by Talati et al.^30^ did not observe significant differences between the effects of plant sterols and stanols on LDL-cholesterol, suggesting that their effects are similar.

The administration of plant sterols/stanols occurred during meals, especially breakfast, lunch and dinner. The studies that used margarine and spread as food matrix or supplementation vehicle oriented its consumption with bread,^16^ ^,^ ^18^ in a sandwich,^15^ ^,^ ^17^ in porridge^18^ or with a hot meal.^17^ It is important that phytosterols be consumed with meals, since esterified phytosterols are hydrolyzed by the cholesterol reductase enzyme in the small intestine in the postprandial period. So, when free, they are available to prevent the incorporation of cholesterol in the micelles.^7^

The vehicles used for supplementation were: tablets, margarine, yogurt, milk, bread, spread, emulsions. Even though all of them significantly reduce LDL-cholesterol, the most significant reduction seems to have occurred with the intake of tablets, whereas bread and emulsions represented inferior reduction. According to a study by Clifton et al.,^31^ which compared the individual efficacy of foods enriched with plant sterols, there was a higher reduction of LDL-cholesterol with skimmed milk, followed by skimmed yogurt; and a lower reduction with bread and cereal, considering the hypothesis that phytosterols may be attached to the core of lipid droplets, thus not being available until the fat is digested. However, the reduction of LDL-cholesterol is also observed in margarine enriched with stanol esters,^15^ ^,^ ^16^ perhaps more than the observed in studies using skimmed milk^25^ and yogurt.^20^ ^,^ ^22^ ^,^ ^24^ Therefore, it is possible that foods enriched with low-fat content phytosterols be equally effective in comparison to those with high fat content. Besides this comparison, there is also the one regarding solid versus liquid meals.^32^ The results obtained in a meta-analysis suggest that high doses of phytosterols in solid foods may have a more effective decreasing effect on LDL-cholesterol than those in liquid meals. However, the discussion about whether or not the type of food (food matrix) impacts its efficiency is still ongoing.^32^

It is also possible that the efficacy of phytosterols may decrease with time of supplementation, as observed in the selected clinical trials in which supplementation was superior than 12 weeks.^14^ ^,^ ^21^ ^,^ ^23^ ^,^ ^24^ The studies that ranged from 8 to 12 weeks presented similar results in the reduction of LDL-cholesterol, and this reduction was higher than 10%, depending on the dose of the supplementation.^14^ ^,^ ^16^ ^,^ ^17^ ^,^ ^19^ ^,^ ^22^ ^,^ ^25^ The only study whose period of intervention lasted for four weeks presented LDL-cholesterol reduction, which, despite being significant, was lower than 10%.^20^

Regarding the beneficial effect of phytosterol supplementation on cholesterolemia, a potential negative effect is phytosterolemia, also known as sitosterolemia, a rare inherited autosomal recessive disorder which is related to mutation in the genes of the cotransporters of phytosterols/cholesterol, ABCG5 and ABCG8. These mutations promote an increase 50 times higher in the circulating concentration of plant sterols, and are associated with early onset atherosclerosis.^33^ ^,^ ^34^ It is important to point out that phytosterol supplementation is contraindicated for the rare patients that present with phytosterolemia,^4^ even though the consumption of foods enriched with phytosterols is associated, in these cases, with a lower increase (about twice as low) in circulating plant sterols.^35^

Another possible side effect is related to the absorption of fat-soluble vitamins and antioxidants. Phytosterols can interfere with the absorption of fat-soluble vitamins^8^ for reducing the levels of LDL-cholesterol, since it is also a carrier of these vitamins. Therefore, if the levels are reduced, transportation will also be reduced.^7^ The serum levels of vitamins A, D and K1, in general, are not affected by the consumption of phytosterols.^35^ However, some of the selected clinical trials suggest that phytosterols may lead to a reduction in the plasmatic concentration of hydrocarbon carotenoids (β-carotene,^14^ ^,^ ^16^ ^,^ ^17^ ^,^ ^19^ α-carotene^14^ ^,^ ^19^ and lycopene^17^), α-tocopherol^16^ ^,^ ^23^ and retinol.^23^ Other studies did not show such reductions.^16^ ^,^ ^17^ ^,^ ^19^ ^,^ ^23^ Despite the concern that fat-soluble vitamins can be reduced by phytosterols, this reduction seems to remain within normality ranges, with no negative implications on health.^36^ Therefore, to prevent any reduction in the serum levels of carotenoids during the intake of phytosterols, the increasing daily consumption of fruits and vegetables rich in carotenoids should be considered.^7^ ^,^ ^19^ ^,^ ^37^

In the pediatric age group, few studies evaluated the effect of phytosterol supplementation regarding lipid profile. However, the studies published until this moment recommend the use of phytosterols as a treatment only for children with hypercholesterolemia who could not reach the ideal levels of LDL-cholesterol after changes in lifestyle.^14^ ^,^ ^15^ ^,^ ^16^ ^,^ ^17^ ^,^ ^18^ ^,^ ^19^ ^,^ ^20^ ^,^ ^21^ ^,^ ^22^ ^,^ ^23^ ^,^ ^24^ ^,^ ^25^ Besides, the approach of phytosterol supplementation may reduce the levels of cholesterol sufficiently, in order to prevent the need for drug therapy.^12^

The clinical trials included in this review presented some limitations, even though they answered the initial question. Some debatable limiting factors were sample size, the short period of follow-up, and the possible confounding variables. Besides, five studies did not report their financing sources,^14^ ^,^ ^17^ ^,^ ^18^ ^,^ ^20^ ^,^ ^21^ and a few reported conflict of interest.^23^ ^,^ ^24^ This review does not present limitation as to the numbers of authors included, considering the strict selection in the choice of studies that responded to the main question.

Despite its limited capacity of reducing the LDL-cholesterol fraction, especially when administered in enriched foods, phytosterol supplementation seems to be of great therapeutic help to reduce cholesterol among children and adolescents with familial hypercholesterolemia, of undetermined etiology or without genetic cause, after the age of 5 years, as established by the Brazilian Society of Cardiology.

It is worth to mention that phytosterols should be used as a form of treatment, instead of prevention, in children/adolescents with hypercholesterolemia who could not achieve the ideal levels of LDL-cholesterol after changes in lifestyle. The administration in meals (lunch and dinner), for a period of 8 to 12 weeks, seems to reach significant reduction in LDL-cholesterol, higher than 10%, together with an adequate diet.

Besides, based on the absence of adverse effects in experimental trials, their use seems to be safe and of good tolerance. Further studies are necessary to analyze the effect of phytosterol supplementation in the long term, in order to verify if its hypocholesterolemic effect is sustained after the intake period.

## References

[1] Lozano P, Henrikson NB, Morrison CC, Dunn J, Nguyen M, Blasi PR (2016). Lipid screening in childhood and adolescence for detection of
multifactorial dyslipidemia: evidence report and systematic review for the
US preventive services task force. JAMA.

[2] Nogueira-de-Almeida CA, Mello ED, Mello PP, Mello PD, Zorzo RA, Ribas-Filho D (2017). Consenso da Associação Brasileira de Nutrologia sobre manejo da
dislipidemia secundária à obesidade infanto-juvenil. Int J Nutrol.

[3] Sociedade Brasileira de Cardiologia (2012). I Diretriz brasileira de hipercolesterolemia familiar
(HF). Arq Bras Cardiol.

[4] Faludi AA, Izar MC, Saraiva JF, Chacra AP, Bianco HT, Afiune A (2017). Atualização da diretriz brasileira de dislipidemias e prevenção
da aterosclerose - 2017. Arq Bras Cardiol.

[5] World Health Organization (1997). Familial hypercholesterolemia (FH). Report of a WHO
consultation.

[6] Sociedade Brasileira de Cardiologia (2005). I Diretriz de prevenção da aterosclerose na infância e na
adolescência. Arq Bras Cardiol.

[7] Malinowski JM, Gehret MM (2010). Phytosterols for dyslipidemia. Am J Health Syst Pharm.

[8] Vuorio A, Kovanen PT (2018). Decreasing the cholesterol burden in heterozygous familial
hypercholesterolemia children by dietary plant stanol esters. Nutrients.

[9] Ras RT, van der Schouw YT, Trautwein EA, Sioen I, Dalmeijer GW, Zock PL (2015). Intake of phytosterols from natural sources and risk of
cardiovascular disease in the European prospective investigation into cancer
and nutrition-the Netherlands (EPIC-NL) population. Eur J Prev Cardiol.

[10] Catapano AL, Graham I, De Backer G, Wiklund O, Chapman MJ, Drexel H (2016). 2016 ESC/EAS guidelines for the management of
dyslipidaemias. Eur Heart J.

[11] Expert Panel on Integrated Guidelines for Cardiovascular Health and
Risk Reduction in Children and Adolescents, National Heart, Lung, and
Blood Institute (2011). Expert panel on integrated guidelines for cardiovascular health
and risk reduction in children and adolescents: summary
report. Pediatrics.

[12] Liberati A, Altman DG, Tetzlaff J, Mulrow C, Gotzsche PC, Loannidis JP (2009). The PRISMA statement for reporting systematic reviews and
meta-analyses of studies that evaluate health care interventions:
explanation and elaboration. PLoS Med.

[13] Santos CM, Pimenta CA, Nobre MR (2007). The PICO strategy for the research question construction and
evidence search. Rev Latino-Am. Enfermagem.

[14] Becker M, Staab D, von Bergmann K (1993). Treatment of severe familial hypercholesterolemia in childhood
with sitosterol and sitostanol. J Pediatr.

[15] Gylling H, Siimes MA, Miettinen TA (1995). Sitostanol ester margarine in dietary treatment of children with
familial hypercholesterolemia. J Lipid Res.

[16] Vuorio AF, Gylling H, Turtola H, Kontula K, Ketonen P, Miettinen TA (2000). Stanol ester margarine alone and with simvastatin lowers serum
cholesterol in families with familial hypercholesterolemia caused by the
FH-North Karelia mutation. Arterioscler Thromb Vasc Biol.

[17] Amundsen AL, Ose L, Nenseter MS, Ntanios FY (2002). Plant sterol ester-enriched spread lowers plasma total and LDL
cholesterol in children with familial hypercholesterolemia. Am J Clin Nutr.

[18] Ketomäki AM, Gylling H, Antikainen M, Siimes MA, Miettinen TA (2003). Red cell and plasma plant sterols are related during consumption
of plant stanol and sterol ester spreads in children with
hypercholesterolemia. J Pediatr.

[19] Amundsen AL, Ntanios F, Put NV, Ose L (2004). Long-term compliance and changes in plasma lipids, plant sterols
and carotenoids in children and parents with FH consuming plant sterol
ester-enriched spread. Eur J Clin Nutr.

[20] Jakulj L, Vissers MN, Rodenburg J, Wiegman A, Trip MD, Kastelein JJ (2006). Plant stanols do not restore endothelial function in pre-pubertal
children with familial hypercholesterolemia despite reduction of low-density
lipoprotein cholesterol levels. J Pediatr.

[21] Matsuyama T, Shoji K, Takase H, Kamimaki I, Tanaka Y, Otsuka A (2007). Effects of phytosterols in diacylglycerol as part of diet therapy
on hyperlipidemia in children. Asia Pac J Clin Nutr.

[22] Guardamagna O, Abello F, Baracco V, Federici G, Bertucci P, Mozzi A (2011). Primary hyperlipidemias in children: effect of plant sterol
supplementation on plasma lipids and markers of cholesterol synthesis and
absorption. Acta Diabetol.

[23] Garaiova I, Muchová J, Nagyová Z, Mišľanová C, Oravec S, Dukát A (2013). Effect of a plant sterol, fish oil and B vitamin combination on
cardiovascular risk factors in hypercholesterolemic children and
adolescents: a pilot study. Nutr J.

[24] Garoufi A, Vorre S, Soldatou A, Tsentidis C, Kossiva L, Drakatos A (2014). Plant sterols-enriched diet decreases small, dense
LDL-cholesyerol levels in children with hypercholesterolemia: a prospective
study. Ital J Pediatr.

[25] Ribas SA, Sichieri R, Moreira AS, Souza DO, Cabral CT, Gianinni DT (2017). Phytosterol-enriched milk lowers LDL-cholesterol levels in
Brazilian children and adolescents: double-blind, cross-over
trial. Nutr Metab Cardiovas.

[26] Plat J, Nichols JA, Mensink RP (2005). Plant sterols and stanols: effects on mixed micellar composition
and LXR (target gene) activation. J Lipid Res.

[27] De Smet E, Mensink RP, Plat J (2012). Effects of plant sterols and stanols on intestinal cholesterol
metabolism: suggested mechanisms from past to present. Mol Nutr Food Res.

[28] Gylling H, Plat J, Turley S, Ginsberg HN, Ellegard L, Jessup W (2014). Plant sterols and plant stanols in the management of
dyslipidaemia and prevention of cardiovascular disease. Atherosclerosis.

[29] Obara CE, Nascimento BL, Danziger C, Mattos FR, Ferreira NA (2018). Propriedades químicas dos estanóis e esteróis
vegetais. Revista Terra & Cultura.

[30] Talati R, Sobieraj DM, Makanji SS, Phung OJ, Coleman CI (2010). The comparative efficacy of plant sterols and stanols on serum
lipids: a systematic review and meta-analysis. J Am Diet Assoc.

[31] Clifton PM, Noakes M, Sullivan D, Erichsen N, Ross D, Annison G (2004). Cholesterol-lowering effects of plant sterol esters differ in
milk, yoghurt, bread and cereal. Eur J Clin Nutr.

[32] Demonty I, Ras RT, van der Knaap HC, Duchateau GS, Meijer L, Zock PL (2009). Continuous dose‐response relationship of the
LDL‐cholesterol‐lowering effect of phytosterol intake. J Nutr.

[33] Renner C, Connor WE, Steiner RD (2016). Sitosterolemia presenting as pseudohomozygous familial
hypercholesterolemia. Clin Med Res.

[34] Buonuomo PS, Iughetti L, Pisciotta L, Rabacchi C, Papadia F, Bruzzi P (2017). Timely diagnosis of sitosterolemia by next generation sequencing
in two children with severe hypercholesterolemia. Atherosclerosis.

[35] Cabral CE, Klein MR (2017). Fitoesteróis no tratamento da hipercolesterolemia e prevenção de
doenças cardiovasculares. Arq Bras Cardiol.

[36] Baumgartner S, Ras RT, Trautwein EA, Mensink RP, Plat J (2017). Plasma fat-soluble vitamin and carotenoid concentrations after
plant sterol and plant sterol consumption: a meta-analysis of randomized
controlled trials. Eur J Nutr.

[37] Noakes M, Clifton P, Ntanios F, Shrapnel W, Record I, McInerney J (2002). An increase in dietary carotenoids when consuming plant sterols
or sterols is effective in maintaining plasma carotenoid
concentrations. Am J Clin Nutr.

